# A Simple Information Criterion for Variable Selection in High‐Dimensional Regression

**DOI:** 10.1002/sim.10275

**Published:** 2024-12-12

**Authors:** Matthieu Pluntz, Cyril Dalmasso, Pascale Tubert‐Bitter, Ismaïl Ahmed

**Affiliations:** ^1^ High‐Dimensional Biostatistics for Drug Safety and Genomics, CESP Université Paris‐Saclay, UVSQ, Université Paris‐Sud, Inserm Villejuif France; ^2^ Laboratoire de Mathématiques et Modélisation d'Évry (LaMME) Université d'Evry Val d'Essonne Évry France

**Keywords:** FWER control, high‐dimensional regression, information criterion, LASSO, pharmacovigilance, variable selection

## Abstract

High‐dimensional regression problems, for example with genomic or drug exposure data, typically involve automated selection of a sparse set of regressors. Penalized regression methods like the LASSO can deliver a family of candidate sparse models. To select one, there are criteria balancing log‐likelihood and model size, the most common being AIC and BIC. These two methods do not take into account the implicit multiple testing performed when selecting variables in a high‐dimensional regression, which makes them too liberal. We propose the extended AIC (EAIC), a new information criterion for sparse model selection in high‐dimensional regressions. It allows for asymptotic FWER control when the candidate regressors are independent. It is based on a simple formula involving model log‐likelihood, model size, the total number of candidate regressors, and the FWER target. In a simulation study over a wide range of linear and logistic regression settings, we assessed the variable selection performance of the EAIC and of other information criteria (including some that also use the number of candidate regressors: mBIC, mAIC, and EBIC) in conjunction with the LASSO. Our method controls the FWER in nearly all settings, in contrast to the AIC and BIC, which produce many false positives. We also illustrate it for the automated signal detection of adverse drug reactions on the French pharmacovigilance spontaneous reporting database.

## 1 Introduction

This work focuses on Information Criteria (IC) in model selection in the context of high‐dimensional regressions, such as those encountered in genomic and drug safety studies. IC are model selection criteria that balance goodness of fit against model complexity (see [1] for the underlying statistical concepts). In practice, an IC selects one model among a family of parametric models by minimizing a function of the number of parameters of the model B, denoted as |B|, its log‐likelihood on the data l(B), and the number of observations n. The function has the following general form:

(1) IC(B)=−2l(B)+pen(|B|)

where pen is the penalty function. Very often, the penalty is a linear function of the model size:

(2) pen(|B|)=w∗|B|

where w is the weight that penalizes the addition of one new parameter into B. The most popular IC are the Akaike Information Criterion (AIC) [2] and the Bayesian Information Criterion (BIC) [3]. They follow the linear form with the following values of w:

- AIC: w=2
- BIC: w=w(n)=log(n)

In high‐dimensional variable selection, we have a regression model with a large number of covariates, p, and we aim to select a sparse sub‐model of it. For combinatorial reasons, the number of sub‐models is too large to allow the computation of each sub‐model's IC. We therefore use a preliminary variable selection algorithm, which yields a smaller family of candidate sub‐models. The LASSO and other penalized regressions with a regularization parameter are examples of such algorithms. Then we select the model with the lower IC among this family. The combination of BIC and preliminary LASSO has been used and compared with other methods, for example in Sabourin et al. [4] and Courtois et al. [5].

However, in high‐dimensional variable selection, the AIC and BIC can yield a large number of false positives [6, 7, 8]. They are not associated with control of multiple testing statistical criteria such as the family‐wise error rate (FWER) or the false discovery rate (FDR).

To address this issue, several authors have proposed information criteria that account for the high‐dimensional nature of variable selection settings by incorporating p in their formula, making the addition of a new parameter into the model more penalized when p is large to account for the large number of candidate models. An overview of them can be found in Bogdan and Frommlet [9].

Foster and George [10] proposed the Risk Inflation Criterion (RIC), which follows the linear form (2) with w=2log(p). Bogdan and Frommlet (2021) introduced the modified AIC (mAIC), where a positive constant is added to the RIC's linear weight: w=2+2log(p/c) where c is a constant for which the authors recommend the value $\frac{1}{2}$.

Other proposals are modifications of the BIC which add terms to the BIC's n‐dependent penalty. Bogdan et al. [11] proposed the modified BIC (mBIC) which—in its version stated in Bogdan and Frommlet—also follows (2), with w=log(n)+2log(p/E) and E a constant that the authors suggest be chosen at E=4. Chen and Chen [6] presented the extended BIC (EBIC), which, in its version stated in Bogdan and Frommlet (2021), follows the general form of an IC (1) with the non‐linear penalty pen(|B|)=log(n)|B|+2(1−κ)logp|B| depending on a constant κ∈[0,1]. There exists also another version of the EBIC, introduced in Chen and Chen [7] which has a more conservative linear penalty, and variations of the mBIC and mAIC with less conservative non‐linear penalties. The various IC may coincide for some values of their constant parameters.

In this work, we propose our own extended version of the AIC, which we call the Extended Akaike Information Criterion (EAIC). This criterion follows the linear form (2). Its w is independent from n (as in the classical AIC) and dependent on p (as in the mBIC and EBIC); it shares these two features with the RIC and the mAIC. We have built the EAIC so that when the p variables are independent, it controls the FWER at a user‐specified level when both n and p are large. The control relies on a property of the asymptotic distribution of the likelihood ratio between two nested models. Section 2 introduces this property, whose detailed proof is in the supporting information, and its meaning in terms of multiple likelihood ratio tests.

Section 3 focuses on variable selection with information criteria. It describes the existing criteria in terms of likelihood‐ratio tests, defines the EAIC based on the results of Section 2, and describes its combination with the LASSO [12]. We conducted a simulation study to assess the performances of the different information criteria, including the EAIC, in both linear and logistic regression models in a variety of high‐dimensional settings, which include a wide range of values of n, p, signal strength, and correlation between the variables. This is described in Section 4. In Section 5, we illustrate our approach by an application in the field of signal detection in pharmacovigilance. This application is based on data from the French national spontaneous reporting database and compares the performances of the various IC using a drug reference set pertaining to drug‐induced liver injuries (DILI) [13].

## 2 FWER Control Bound in Multiple Independent Likelihood Ratio Tests

This section presents a property of likelihood‐ratio tests of the insertion of one more parameter to a parametric model. This property makes it possible to approximately control the FWER in multiple independent tests by setting the test's critical value at a simple function of the approximate number of tests and of the desired FWER.

### 2.1 Rationale and Notations

Consider a statistical model with log‐likelihood l(θ;X). Let $\Theta_{0}$ be a subspace of the parameter space having dimension d and $\Theta_{1}$ a subspace having dimension d+1, containing $\Theta_{0}$. Let $l_{0}$ be the maximum of log‐likelihood over $\Theta_{0}$, $l_{1}$ the maximal log‐likelihood over $\Theta_{1}$, and $\Delta l=l_{1}-l_{0}$. Let $H_{0}$ be the hypothesis that the true parameter vector is in $\Theta_{0}$ and $H_{1}$ be the alternative hypothesis that it is in $\Theta_{1}\setminus \Theta_{0}$.

Wilks' theorem [14] asserts that under $H_{0}$, 2Δl has asymptotically a $\chi^{2}$ distribution with one degree of freedom, that is, that Δl follows asymptotically a gamma distribution of parameters $\left(\frac{1}{2},1\right)$. Let f be the probability density function of this distribution, and F its cumulative distribution function. In a likelihood‐ratio test, the statistic Δl is used to test $H_{0}$ against $H_{1}$. A procedure that rejects $H_{0}$ in favor of $H_{1}$ if Δl>x, for some bound x, has an asymptotic probability 1−F(x) of incorrectly rejecting $H_{0}$ when it holds.

Suppose now that there are q subspaces of parameters $\Theta_{1}^{1},\ldots ,\Theta_{1}^{q}$ of dimension d+1, all containing $\Theta_{0}$. Let $H_{i}$ be the hypothesis that the true parameter vector is in $\Theta_{1}^{i}\setminus \Theta_{0}$, $l_{1}^{i}$ the maximum of the log‐likelihood over $\Theta_{1}^{i}$, and $\Delta l^{i}=l_{1}^{i}-l_{0}^{i}$.

$H_{0}$ is tested against each $H_{i}$, for i=1,…,q with a procedure that rejects $H_{0}$ in favour of $H_{i}$ if $\Delta l^{i}>x$ for some bound x which does not depend on i. These tests are a tool of model selection: if $H_{0}$ is rejected for none of the i, then the model where the parameters are in $\Theta_{0}$ is selected. If it is rejected at least once, then a model with more parameters will be selected. We want to control the probability of this event under $H_{0}$.

We assume that the q likelihood‐ratio tests are independent, that is, the $\Delta l^{i}$ are independently distributed. Then, under $H_{0}$, the asymptotic probability that the correct model is selected (i.e., that none of the q tests reject $H_{0}$) is $F^{q}(x)=F{\left(x\right)}^{q}$. $F^{q}$ is the asymptotic cumulative distribution function of $\max(\Delta l^{1},\ldots ,\Delta l^{q})$. We will prove an asymptotic property of $F^{q}$ for large q, which translates into FWER control.

### 2.2 A Simple Case

For all α∈(0,1), the sequence ${\left(x_{q,\alpha }\right)}_{\;q\geq 2}$ with:

(3) $$x_{q,\alpha }=\log q-\frac{1}{2}\log\log q-\log\left(-\log(1-\alpha )\right)-\frac{1}{2}\log\pi$$

has the property that:

$$\underset{q\to \infty }{\lim}F^{q}(x_{q,\alpha })=1-\alpha$$

In other words, when performing a large number q of independent likelihood‐ratio tests of $H_{0}$ against $H_{i}$ for i=1,…,q, if the $\Delta l^{i}$ follow their asymptotic distribution, then rejecting $H_{0}$ in favor of $H_{i}$ if $\Delta l^{i}>x_{q,\alpha }$ controls the family‐wise error rate at level α.

Given the definition (3), the result has its simplest form when $\alpha =\alpha_{0}=1-e^{-\frac{1}{\sqrt{\pi }}}\approx 0.43118$. The bound $x_{q,\alpha_{0}}=\log q-\frac{1}{2}\log\log q$ controls the family‐wise error rate at level $\alpha_{0}$.

### 2.3 General Case

In practice, the critical value x must not vary with the exact number of tests q. This is because, as detailed in Section 3, using an information criterion amounts to doing several multiple testing procedures where q varies a little but x stays the same across the families of tests. Therefore, we replace q by a proxy quantity approximately equal to it, $q^{\prime }$, in the definition of x. We will prove that:

For all α∈(0,1), for every sequence $q^{\prime }(q)$ (denoted just $q^{\prime }$ in the following) such that $q^{\prime }{∼}_{\infty }q$, the sequence $x_{\cdot ,\alpha }$ defined in (3) satisfies:

$$\underset{q\to \infty }{\lim}F^{q}(x_{q^{\prime },\alpha })=1-\alpha$$

This means that when performing a large number q of independent likelihood‐ratio tests where the likelihood ratios follow their asymptotic distribution, for all values of $q^{\prime }$ approximately equal to q, the bound $x_{q^{\prime },\alpha }$ controls the family‐wise error rate at level α.

We prove this property (and consequently its sub‐case stated in Section 2.2) in subsection 1 of the supporting information.

Therefore, for any family of independent tests when both the number of observations n and of tests q are large, we can reasonably suppose that the family‐wise error rate is controlled at this level (although the theorem above does not deal with the asymptotics in n: it assumes that n is large enough to use the asymptotic distribution of the $\Delta l^{i}$).

## 3 Variable Selection in High‐Dimensional Regressions

We use the result of Section 2 to quantify the FWER of information‐criteria‐based sparse model selection methods in the simple case of comparing a model with all those having one more parameter, and then to build an FWER‐controlling information criterion, the EAIC. We focus on generalized linear regression models, although the same reasoning would work for any large parametric model.

### 3.1 AIC and BIC

Consider a generalized linear model:

$$h\left(E[Y]\right)=\sum_{j=1}^{p}\beta_{j}X_{j}$$

p is assumed to be a large integer, the $X_{j}$ are assumed to be independent, and the $\beta_{j}$ are assumed to be zero when j is not part of some unknown sparse subset $A^{*}$ of {1,…,p}. The goal of variable selection is to determine $A^{*}$ based on observations of Y and the $X_{j}$. For every subset A of {1,…,p}, it is possible to compute the log‐likelihood l(β) of the following sub‐model (also called model A):

$$h\left(E[Y]\right)=\underset{j\in A}{\sum }\beta_{j}X_{j}$$

Then one model is selected by minimizing an information criterion depending on l(A) and on the number |A| of variables in A, such as the AIC. It may be interpreted in terms of a likelihood‐ratio test. Let B be a subset containing A and having one more variable than A; then

AIC(B)−AIC(A)=2−2Δl

Therefore, when minimizing the AIC over A and B, B is selected if Δl>1, which has an asymptotic probability of 1−F(1)≈0.1573 if $A^{*}\subset A$. The AIC is therefore a way of setting the type 1 error rate at a fixed level.

Nonetheless, in this high‐dimensional regression problem, the use of the AIC (or any other similar criterion) is akin to multiple likelihood‐ratio testing. Since p−|A| variables are not included in A, there are p−|A| subsets B that contain A and have one more variable than A. When we compare the AIC of all these subsets, the probability of selecting one of them rather than A if A is the true model (family‐wise error rate) is $1-F{\left(1\right)}^{p-|A|}$ (assuming that each $\Delta l^{i}$ follows the cumulative distribution function F and that they are independent of each other), which approaches 1 when p is large.

The test independence condition is seldom exactly verified. It is closer to being verified when the $X_{j}$ are not, or are weakly, correlated with each other. A nonzero correlation between the $X_{j}$ would typically create a positive correlation between the corresponding $\Delta l^{j}$. The probability of wrongly selecting a model larger than A when A is the true model would then be lower than in the independent case, but it would still converge to 1.

If the BIC is used rather than the AIC, then if B has one more variable than A:

BIC(B)−BIC(A)=log(n)−2Δl

Assuming again that each $\Delta l^{i}$ follows F and that the tests are independent, the family‐wise error rate is:

$${\text{FWER}}_{\text{BIC}}(n,p)=1-F{\left(\frac{1}{2}\log n\right)}^{p-|A|}$$

If the models that we are comparing are sufficiently sparse that |A|≪nlog(n), then:

$${\text{FWER}}_{\text{BIC}}(n,p)\approx 1-e^{-\frac{p}{\sqrt{\pi n\log(n)/2}}}$$

If n goes to infinity while p is of the order of n or smaller, the BIC's family‐wise error rate converges to 0. However, as this is a high‐dimensional regression, we cannot make this hypothesis. For example, if p is of the order of $n^{k}$ for some k larger than 1, the BIC's family‐wise error rate converges to 1. Variable selection based on the BIC does not asymptotically bound the family‐wise error rate in general.

### 3.2 Extended AIC

The results of Section 2 suggest a way of controlling the family‐wise error rate while using a criterion similar to the AIC. For every α∈(0,1), for every subset A of {1,…,p}, let us define the extended AIC:

$${\text{EAIC}}_{\alpha }(A)=2x_{p,\alpha }|A|-2l(A)$$

where the weight $2x_{p,\alpha }$ is, as defined in (3):

$$2x_{p,\alpha }=2\log p-\log\log p-2\log\left(-\log(1-\alpha )\right)-\log\pi$$

Unlike the AIC and BIC, the EAIC is not a function of the intrinsic characteristics of the model A only. It also takes into account the fact that A is considered along with all the other sub‐models of a p‐dimensional regression model.

Like the AIC and BIC, the definition translates into the following difference for two nested models A and B, where B has one more parameter:

$${\text{EAIC}}_{\alpha }(B)-{\text{EAIC}}_{\alpha }(A)=2x_{p,\alpha }-2\Delta l$$

B is chosen over A if and only if $\Delta l>x_{p,\alpha }$. Therefore, by minimizing the EAIC among all the p−|A| subsets containing the true active subset A and having one more variable than A, we have the situation in Section 2.3 where q=p−|A| and $q^{\prime }=p$. Since the setting is high‐dimensional (large p and large n) and the subsets that we are comparing are relatively sparse (|A|≪p), the asymptotic setting of Section 2.3 (q→∞ and $\;q^{\prime }∼q$) is well approached, and we make the assumption that the $\Delta l^{i}$ follow their asymptotic distribution with cumulative distribution function F. Therefore, the family‐wise error rate approaches

$$1-F_{\max}^{p-|A|}(x_{p,\alpha })\approx \alpha$$

#### 3.2.1 Coincidence With Other Information Criteria

For some values of α or p, the EAIC coincides with other information criteria. It coincides with the RIC (of weight 2logp) when:

loglogp+2log(−log(1−α))+logπ=0

that is,

$$\alpha =\alpha_{\text{RIC}}(p)=1-e^{\frac{-1}{\sqrt{\pi \log p}}}$$

The term $1/\sqrt{\pi \log p}$ (which is equivalent to $\alpha_{\text{RIC}}(p)$ at large p) has been noted in Bogdan and Frommlet [9] to be interpretable as the RIC's family‐wise test level. $\alpha_{\text{RIC}}(p)$ decreases at a very slow rate, with $\alpha_{\text{RIC}}(100)\approx 0.231$, $\alpha_{\text{RIC}}(10^{4})\approx 0.186$, and $\alpha_{\text{RIC}}(10^{6})\approx 0.152$.

The EAIC also coincides with the mAIC (of weight 2logp−2logc+2) when:

loglogp+2log(−log(1−α))+logπ=2logc−2

that is,

$$\alpha =\alpha_{\text{mAIC}}(p,c)=1-e^{\frac{-c}{e\sqrt{\pi \log p}}}$$

or

$$c=c_{\text{EAIC}}(p,\alpha )=-2e\sqrt{\pi \log p}\log(1-\alpha )$$

$\alpha_{\text{mAIC}}$ decreases in p at the same slow rate as $\alpha_{\text{RIC}}$, asymptotically multiplied by the constant c/e. At the recommended $c=\frac{1}{2}$, $\alpha_{\text{mAIC}}(100,\frac{1}{2})\approx 0.0472$, $\alpha_{\text{mAIC}}(10^{4},\frac{1}{2})\approx 0.0336$, and $\alpha_{\text{mAIC}}(10^{6},\frac{1}{2})\approx 0.0275$.

#### 3.2.2 Simplification and Approximation of the EAIC

We noted in Section 2.2 that $x_{p,\alpha }$ simplifies when $\alpha =\alpha_{0}=1-e^{-\frac{1}{\sqrt{\pi }}}$. Therefore, the EAIC has the following simpler version, whose family‐wise error rate approaches $\alpha_{0}\approx 0.4312$:

$${\text{EAIC}}_{\alpha_{0}}(A)=\left(2\log p-\log\log p\right)|A|-2l(A)$$

Furthermore, owing to the asymptotics of $x_{p,\alpha }$ when α→0, the EAIC has the following approximation at small values of α:

$${\text{EAIC}}_{\alpha }(A)\approx \left(2\log p-\log\log p-2\log\alpha -\log\pi \right)|A|-2l(A)$$

### 3.3 Model Selection Procedure With the EAIC

We have defined a new information criterion that can be computed for any given submodel. Like the others IC, it could in principle be used to select a sparse submodel of interest by computing it for every submodel and choosing the one with the lowest IC. Although the FWER properties of Sections 3.1 and 3.2 do not automatically translate into equivalent results for this procedure, they are a good indication of the tendency of information criteria to produce false positives.

In practice, as a consequence of the high dimension of the regression problem, the number of subsets of variables is actually too large to compute the likelihood of each of them. A solution is to use a pre‐selection procedure that yields a smaller family of sparse candidate models that explain the outcome particularly well and then to minimize the IC among these models. This is a computationally feasible proxy for minimizing the IC among all the sub‐models. The pre‐selection procedure we used is the LASSO penalized regression [12], where the variations of the regularization parameter deliver a list of sparse models, the LASSO path.

Note that the log‐likelihood used in the information criteria is non‐penalized. This is because Wilks' theorem describes the distributions of the maximal loglikelihoods of nested models. Comparing their maximal penalized log‐likelihoods, or the log‐likelihoods at the parameters where the penalized log‐likelihoods are maximal, would introduce an extra term in the Δl due to the difference in penalization; the sparser, higher‐ λ models shrink their LASSO estimates of β more, which puts their log‐likelihood further from its maximal value. Therefore, to combine the LASSO and an IC, it is necessary to fit each of the models selected by the LASSO without penalty, and not just to reuse the models fitted with the LASSO's penalty.

## 4 Simulation Study

We conducted two simulation studies. The first one aimed to assess the FWER control given by the EAIC in a simple framework, where it tests all the models having one active variable against the null one (with no active variables) under the null hypothesis. The second one explored the performance of the LASSO‐based complete model selection procedure of Section 3.3 in settings with active variables. The R code of our simulation studies is provided in our supporting information.

### 4.1 FWER Control Under the Null Hypothesis

We used the EAIC to compare the null model $h\left(E[Y|X]\right)=\beta_{0}$ with the p models with one active variable $h\left(E[Y|X]\right)=\beta_{0}+\beta_{j}X_{j},\;\;j=1,\ldots ,p$. We simulated data under the null model, computed each model's EAIC, and selected the model with one active variable having the lowest EAIC if it was also lower than the null model's EAIC. This amounts to multiple likelihood‐ratio tests where the EAIC performs multiple testing corrections. For a given setting, we assessed the control of the FWER by computing the estimated FWER, which is the proportion of simulated datasets where a non‐null model is selected.

This can be viewed as the first step of the complete model selection procedure: we minimize the EAIC among the models of size only 0 or 1 instead of a whole sequence of models. Unlike in Section 3.3, the minimization is done across all these candidate models without using the LASSO or any other pre‐selection method. For computational reasons, the exception to this were the settings where $p=10^{4}$, where we computed the EAIC of the model including $X_{j}$ only for the j where $\left|Cor(Y,X_{j})\right|$ was at least 90% of its maximal value across all j.

#### 4.1.1 Data Simulation

We simulated 1000 datasets from each of 30 settings, described by the following parameters:

- The regression model family: linear or logistic.
- The number of observations is $n=10^{2},10^{3}$, or $10^{4}$.
- The number of regressors $p=10^{2},10^{3}$, or (only in non‐correlated settings) $10^{4}$.
- The correlation matrix used to simulate the regressors is a Toeplitz matrix $\rho_{\left(i,j\right)}=\rho^{\left|i-j\right|}$, with ρ=0 or ρ=0.5. For computational reasons, settings where $\left(p=10^{4},\rho =0.5\right)$ were excluded.

The regressors $X_{j}$ were drawn following a standard normal distribution. The outcome Y was drawn independently from the $X_{j}$, following a standard normal distribution in the linear settings and a Bernoulli distribution with probability 0.5 in the logistic settings. We used the EAIC with α=0.05.

#### 4.1.2 Simulation Results

As shown in Table 1, the FWERs that we observed in the 30 settings ranged between 0.029 and 0.055, in agreement with the targeted level.

**TABLE 1:** Null hypothesis simulation study: observed FWER by setting, averaged on 1000 simulations.

Parameters			$p=10^{2}$	$p=10^{3}$	$p=10^{4}$
Linear	ρ=0	$n=10^{2}$	.046	.045	.055
		$n=10^{3}$	.030	.030	.039
		$n=10^{4}$	.043	.040	.049
	ρ=0.5	$n=10^{2}$	.039	.049	
		$n=10^{3}$	.033	.050	
		$n=10^{4}$	.032	.048	
Logistic	ρ=0	$n=10^{2}$	.047	.045	.049
		$n=10^{3}$	.037	.037	.036
		$n=10^{4}$	.029	.046	.039
	ρ=0.5	$n=10^{2}$	.039	.050	
		$n=10^{3}$	.036	.044	
		$n=10^{4}$	.029	.050	

### 4.2 Simulation of the Full Procedure

#### 4.2.1 Design

##### 4.2.1.1 Data Simulation

We compared the performance of the EAIC with that of other Information Criteria‐based variable selection methods on 308 different settings of the generalized linear model $h\left(E[Y|X]\right)=\beta_{0}+X\beta$. The settings were determined by:

- The regression model family: linear or logistic.
- The number of observations is $n=10^{2},10^{3}$, or $10^{4}$.
- The number of regressors $p=10^{2},10^{3}$, or (only in non‐correlated settings where $n<10^{4}$) $p=10^{4}$.
- The correlation matrix used to simulate the regressors is a Toeplitz matrix $\rho_{\left(i,j\right)}=\rho^{\left|i-j\right|}$, with ρ=0 or (only where $p<10^{4}$) ρ=0.5.
- The empirical signal‐to‐noise ratio. This quantity was based on the signal‐to‐noise ratio used in Sabourin et al. 2015, although unlike them we used an empirical version, which is defined for every generalized linear model. It is the ratio of the empirical variance of the signals ($E_{\beta }[Y_{i}|X],\;i=1,\ldots ,n$) over the empirical mean of the noises' variances (${\mathrm{Var}}_{\beta }(Y_{i}|X),\;i=1,\ldots ,n$):
$$\text{SNR}(X,\beta )=\frac{\frac{1}{n-1}\sum_{i=1}^{n}{\left(E_{\beta }[Y_{i}|X]-\frac{1}{n}\sum_{i=1}^{n}E_{\beta }[Y_{i}|X]\right)}^{2}}{\frac{1}{n}\sum_{i=1}^{n}{\mathrm{Var}}_{\beta }(Y_{i}|X)}$$
  A larger SNR means a more easily observable impact of each active $X_{j}$ on Y. Variable selection is expected to be more efficient in settings with large SNR. We set SNR(X,β)=0.005,0.01,0.02,0.05,0.1,0.2,0.5,1,2,5or10.

We simulated 1000 datasets in each of these settings. In each simulation, we first drew the regressor matrix X following a standard normal distribution (with or without correlation depending on the setting). Then we drew 10 active regressors uniformly among the p regressors. We determined their coefficients $\beta_{j}$ with the following algorithm:

- Draw non‐normalized coefficients ${\tilde{\beta }}_{j}$ at random with $P({\tilde{\beta }}_{j}>0)=\frac{1}{2}$ and $\left|{\tilde{\beta }}_{j}\right|$ following a uniform distribution on (0.25,1), each independently from one another and from their sign;
- Define $\beta_{0}$ and $\beta_{j}$ as the normalized coefficients: $\beta_{j}=k(X){\tilde{\beta }}_{j}$ where we computed k(X) and $\beta_{0}$ via numerical equation solving (function multiroot in the R package rootSolve version 1.8.2.3) [15] so that SNR(X,β) is the desired value.

We then simulated the simulation's outcome vector y following the generalized linear model based on the simulated X and β. In the linear model, ${\mathrm{Var}}_{\beta }(Y|X)=1$.

Due to computational limitations in the simulation of X, the settings where $\left(p=10^{4},\rho =0.5\right)$ or $\left(p=10^{4},n=10^{4}\right)$ were not included.

##### 4.2.1.2 Method Comparison

For each simulated dataset, we ran the LASSO algorithm using R's glmnet package version 4.1‐2 [16] with the default parameters and at most 100 degrees of freedom (parameter dfmax). We applied seven different variable selection methods, which all consist in choosing one of the models appearing on the LASSO path. Six methods select the model that minimizes an IC:

- the AIC
- the BIC
- the EBIC at κ=1
- the mAIC at c=1/2
- the mBIC at E=4
- the EAIC at α=0.5
- and the EAIC at α=0.05.

These methods require fitting the non‐penalized version of each of the models on the LASSO path and then using the resulting log‐likelihood to compute the criteria. The seventh method, which we call “oracle”, uses the information that there are 10 active variables. It consists in selecting the first model with at least 10 variables on the LASSO path. Its purpose is to illustrate how accurate variable selection can be expected for each setting.

We computed estimates of the family‐wise error rate (FWER), the false discovery rate (FDR), and the sensitivity of each of these methods for each of the 308 settings by averaging over the 1 000 simulated datasets. The sensitivity is the expected proportion of selected variables among the active variables; since all settings have 10 active variables, we indicate the average number of those true positives, which is proportional to the estimated sensitivity.

#### 4.2.2 Results

For each combination of n, p, ρ, and model family, we plotted the graph of each method's FWER (Figures 1, 2, 3, 4), average number of true positives (Figures S1–S4) and FDR (Figures S5–S8) varying with the SNR.

**FIGURE 1:**
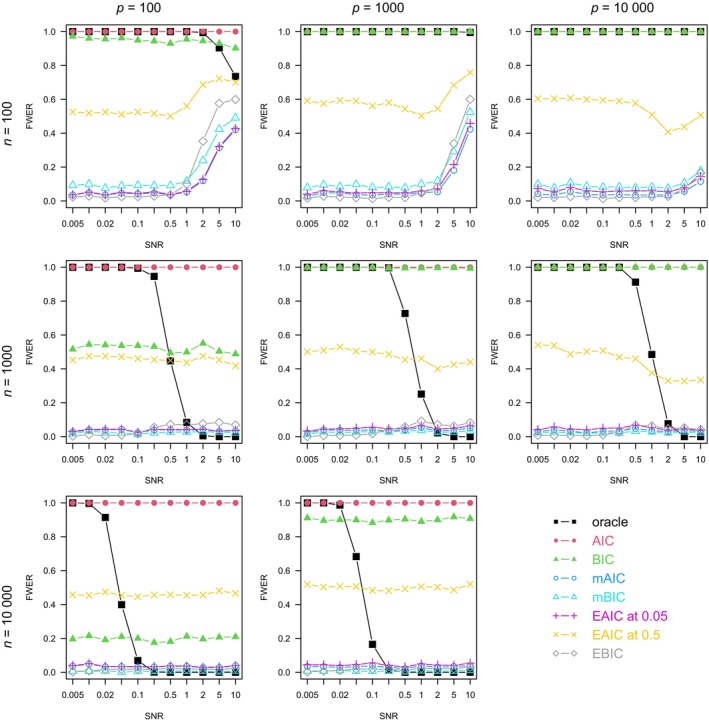
Full procedure simulation study: FWER by setting, averaged on 1000 simulations. Linear model, ρ=0.

**FIGURE 2:**
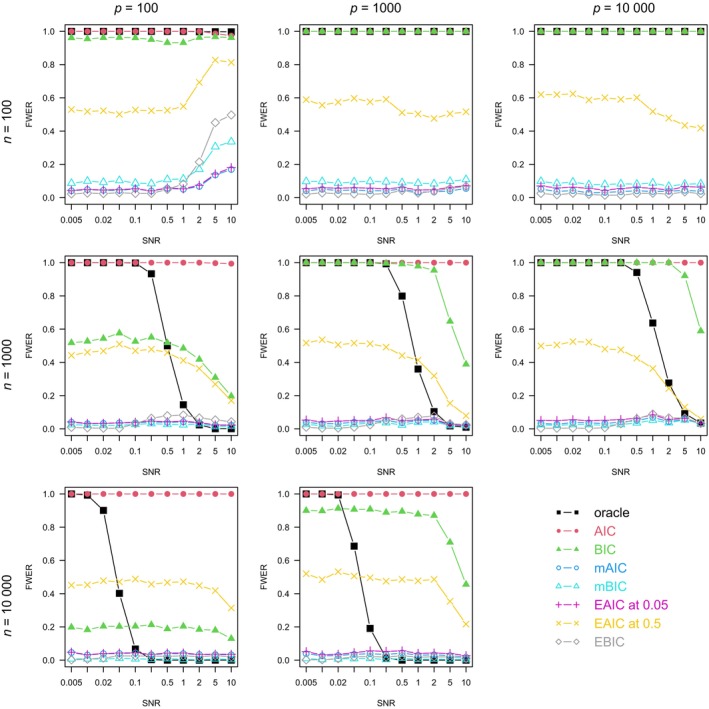
Full procedure simulation study: FWER by setting, averaged on 1000 simulations. Logistic model, ρ=0.

**FIGURE 3:**
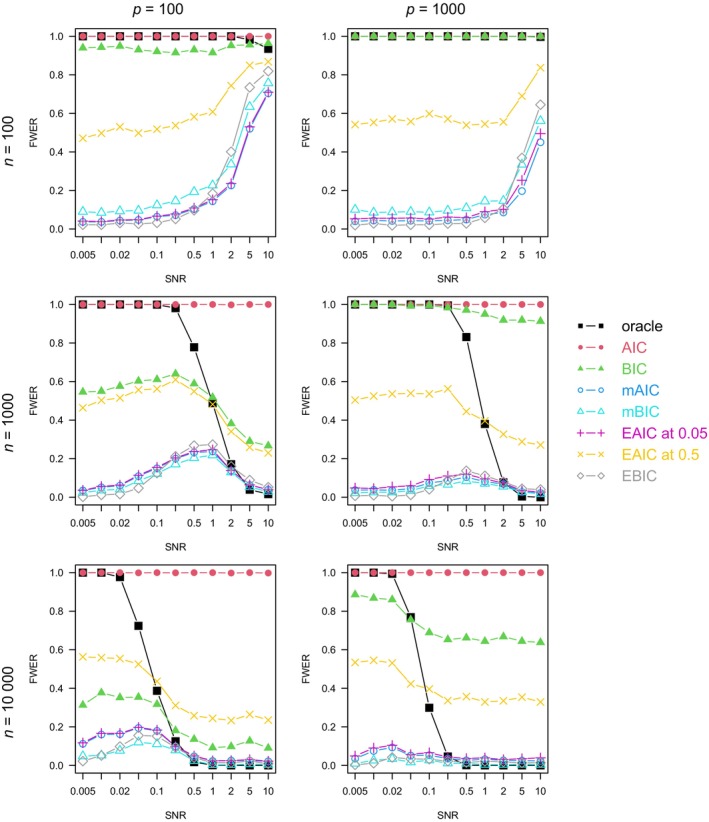
Full procedure simulation study: FWER by setting, averaged on 1000 simulations. Linear model, ρ=0.5.

**FIGURE 4:**
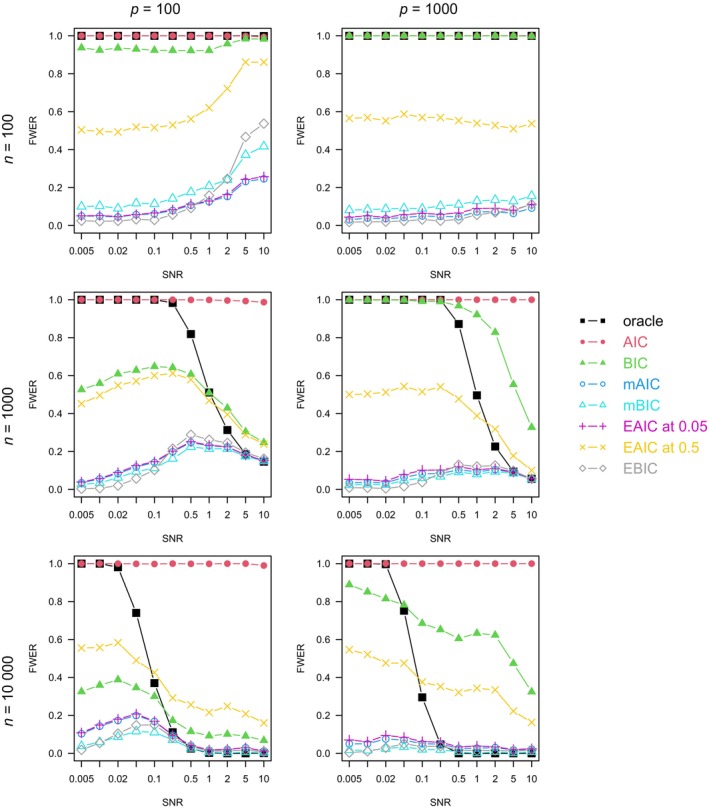
Full procedure simulation study: FWER by setting, averaged on 1000 simulations. Logistic model, ρ=0.5.

In Figures 1, 2, 3, 4, the oracle's FWER indicates the models in which it is possible to capture the exact 10 active variables by stopping at a node of the LASSO path. When n=100 or the SNR is low, it is almost never possible since the oracle's FWER is at or close to 1. In non‐correlated settings with n≥100 and sufficiently high SNR (Figures 1 and 2), the oracle's FWER approaches 0, which shows that the correct set of 10 variables is on the LASSO path.

The AIC's FWER, almost always at 1, shows that this method almost always produces false positives.

In non‐correlated settings (Figures 1 and 2), the BIC's FWER tends not to depend on the SNR (except in high‐SNR, high‐ n, logistic models where there is a decrease) but is highly dependent on both n and p. In linear settings with few observations (n=100) or many variables (p≥1000), it is at or close to 1, meaning that the BIC also almost always produces false positives. Only at large n and small p does the BIC have a moderate FWER.

Figures 1 and 2 show that in accordance with its theoretical basis, the extended AIC approximately controls the FWER at the desired level in most non‐correlated settings. The only exceptions are the setting with few observations (n=100), high SNR, and moderate number of variables (p≤1000 in the linear model, p=100 in the logistic one). In those settings, the high SNR and moderate p imply that a large proportion of the active variables are captured (as seen on the sensitivity curves of Figures S1 and S2). Therefore, the EAIC compares models of size of the order of 10. At this size, the convergence in distribution guaranteed by Wilks' theorem is slower than when comparing models of very small size. Therefore, at n=100, the asymptotic regime is not reached, so the theoretical basis for FWER control by the EAIC does not hold. Moreover, in non‐correlated settings, the EAIC's FWER does not drop much below its nominal value except in high‐SNR logistic models (Figure 2).

The other information criteria that penalize for p—mBIC, mAIC, and EBIC—generally have a low FWER, except in the same settings where the EAICs' FWER control fails: n=100, high SNR, and moderate number of variables. They exhibit patterns similar to the EAIC at 0.05's FWER, although the mBIC's and the EBIC's FWER show more variation, being sometimes close to 0 and having higher maximal values. The mAIC has a FWER always slightly below that of the EAIC at α=0.05 with a bit more divergence at large p, in accordance with their coincidence described in Section 3.2.1.

In correlated settings (Figures 3 and 4), the FWER varies somewhat more with the SNR for all methods except the AIC (which has a FWER always close to 1). When n=100, the FWERs have a similar pattern to those observed in non‐correlated settings, with an even higher spike at high SNR and a moderate number of variables. When n≥1000, there is some deviation from the FWER control, which was observed in non‐correlated settings. Over all the n≥1000,ρ=0.5 settings, the EAIC at α=0.5 has a maximal FWER of 0.612, the EAIC at α=0.05 has 0.251, the EBIC has 0.288, the mAIC has 0.247, and the mBIC has 0.224. In the most favorable correlated settings, at large n and SNR (when the correct model is almost always on the LASSO path, as shown by the oracle's FWER approaching 0), the EAICs control the FWER at or below its nominal level.

Figures S1–S4 show the number of true positives captured by each method in each setting, which is 10‐fold their sensitivities since all settings are simulated with 10 active variables. The sensitivities increase with the SNR and with n but decrease with p. The most conservative methods in terms of FWER are also the least sensitive. The EAIC at α=0.05, mAIC, mBIC, and EAIC have sensitivities close to each other's. However, there are two types of settings where two groups differ in sensitivity:

- In the large n, low SNR settings, EAIC and mAIC have a higher sensitivity than EBIC and mBIC. They also have a slightly higher FWER, but the EAIC still controls its FWER at the nominal level.
- On the contrary, in the small n, small p, high SNR settings, EBIC and mBIC have a higher sensitivity than EAIC and mAIC. This comes with the price of higher FWERs, which reach their highest values in these settings.

The curves of FDR (Figures S5–S8 in the supporting information) show the same hierarchy of methods, with the AIC being the least conservative and the EBIC being on average the most conservative. While the mAIC, EAIC at α=0.05, and mBIC have a higher FDR than the EBIC averaged across the settings, their FDR is more stable, being respectively at most 0.075, 0.083, and 0.107 in non‐correlated settings and respectively at most 0.125, 0.126, and 0.141 in correlated settings, while the EBIC reaches 0.128 in a non‐correlated setting and 0.171 in a correlated setting. Among those four, the mBIC has the highest FDR when n is small. The EAIC at α=0.5, although generally not conservative, has a FDR that never approaches 1 (maximal FDR equal to 0.623), as opposed to the BIC, which has a FDR close to 1 in small n, large p, low SNR settings.

## 5 Application to Pharmacovigilance Data

To illustrate the behavior of the variable selection methods on real data, we applied them to the French pharmacovigilance database (BNPV). We used the same data preprocessing as described in [5], yielding a database of n=452914 spontaneous reports of adverse drug reactions from 1 January 2000 to 29 December 2017 with 6617 different adverse events (coded according to the Preferred Term level of the Medical Dictionary for Regulatory Activities, MedDRA) and p=1692 different drugs (coded with the 5th level of the Anatomical Therapeutic Chemical hierarchy) reported at least 10 times. We focused on a binary outcome, the adverse event Drug‐Induced Liver Injury (DILI), and we used a logistic regression model based on drugs as binary covariates. A pharmacovigilance signal was defined as a variable that is selected with a positive estimated coefficient.

To assess the performances of the methods, we used the same reference set of pharmacovigilance signals as [5] pertaining to DILI [13]. It includes 203 negative controls (drugs known not to be associated with DILI) and 133 positives (drugs known to be associated with DILI).

We implemented six methods that minimize an information criterion on the LASSO path: the AIC, the BIC, the EAIC at various levels of α, the EBIC, the mBIC, and the mAIC. Table 2 shows the results of these methods, including the EAIC at 50%, 10%, and 5%. The False Discovery Proportion (FDP), specificity, and sensitivity were computed on drugs with known status. The results are shown in Table 2. As in most simulation settings, the AIC generated the most signals. The mBIC generated the least.

**TABLE 2:** Performance of each method on the BNPV dataset in terms of number of pharmacovigilance signals (variables positively associated with DILI), False Discovery Proportion (FDP), specificity and sensitivity.

Methods	Signals	Signals with known status	False positives	FDP (%)	Specificity (%)	Sensitivity (%)
AIC	187	69	5	7.2	97.5	48.1
EAIC at 50%, BIC	170	65	5	7.7	97.5	45.1
EAIC at 10%mAIC	150	59	4	6.8	98.0	41.4
EAIC at 5%, EBIC	142	55	2	3.6	99.0	39.8
mBIC	118	48	2	4.2	99.0	34.6

The sensitivities of the methods reflect this hierarchy, ranging from 48.1% for the AIC to 34.6% for the mBIC. Several pairs of criteria selected the same model and therefore had the same characteristics on this dataset. This is the case of the EAIC at α=0.5 with the BIC (the level at which the EAIC and BIC were mathematically equivalent being α≈0.406 for these values of n and p), the EAIC at α=0.1 with the mAIC, and the EAIC at α=0.05 with the EBIC. With only two known false positives, these last two had the best specificity together with mBIC (99.0%), and the single best FDP (3.6%).

## 6 Discussion

This work focuses on high‐dimensional sparse model selection using information criteria. Compared to variable selection based on univariate models, multivariate model selection is necessary to avoid producing false positives or false negatives when the regressors are correlated. Consequently, although an independence hypothesis was involved in the design of the method we propose, it was important to also measure its properties in settings with correlated regressors.

The AIC and BIC, which are classical IC, are often used for high‐dimensional sparse model selection. In high dimensions, they do not have consistency properties and tend to select models with many false positives, as confirmed by our extensive simulation study. However, we show that limiting the number of false positives while using an IC in high dimension is possible. This is done by choosing a criterion that adjusts for the dimensionality: either the extended BIC, the modified BIC, the modified AIC, or one of their variants; or our proposal, the extended AIC.

Unlike the other information criteria, the EAIC is designed to control the family‐wise error rate at a specified level. Our mathematical result suggests that it achieves this when the candidate variables are not correlated and n and p are large enough for asymptotic approximations to hold. In practice, the simulations show that the EAIC's FWER is indeed close to its specified level in nearly all non‐correlated settings (across wide ranges of n and p, in both linear and logistic models), the only exceptions being when both n and p are small in conjunction with the active variables having a strong effect.

Moreover, the “modified” or “extended” criteria achieve satisfactory FDR performance in contrast with the classical criteria. Although we do not provide mathematical results supporting this claim, our simulations show that while both the AIC and BIC have complete failure modes (settings where their FDR approaches 1), this is not the case of the criteria that penalize for p. Even in settings where the candidate variables are correlated, their FDR is at most about 0.6 (EAIC at α=0.5) or always below 0.2 (all the other IC which correct for p).

The various IC used in combination with the LASSO are deterministic, simple, and fast methods. They only require fitting the LASSO once and then fitting a sequence of low‐dimensional, non‐penalized regressions without having to re‐sample or simulate data as opposed to (among others) cross‐validation. Although our simulations focused on the LASSO, another pre‐selection procedure can be used in its place as long as it provides a small family of sparse candidate models. This is the case of other penalized regressions such as SCAD [17] and elasticnet [18]. It has been observed that the LASSO path can include avoidable false positives early on [19], which may induce to combine the IC with another pre‐selection method.

Compared to the other information criteria, the EAIC's parametrization is directly interpretable, as α is the FWER that one obtains under asymptotic and test independence assumptions—which is in practice the FWER observed in most non‐correlated settings. While the mAIC controls the FWER at known values, which slowly decrease with p [9], the EAIC is the only information criterion that controls the FWER at any level that the user may input.

The EAIC uses the number p of covariates by interpreting it as an approximation of the number of likelihood‐ratio tests that IC optimization implicitly performs and by making the strong assumption that those tests are independent. Since the tests are in practice non‐independent, mostly because of the correlation between covariates, it can be desirable to replace p with a measure of the effective number of independent tests to which the p non‐independent tests are approximately equivalent when correcting for multiple testing. The notion is used in multiple testing in genomic analysis, with varying definitions [20, 21]. In the context of genomics, Bogdan et al. [22] have proposed an α‐dependent effective number of tests, which they use in the mBIC, and might also work with the EAIC.

Therefore, we recommend that statistical practitioners who need a simple method for high‐dimensional variable selection based on the LASSO or another pre‐selection method and who want to make sure that they do not mostly select false positives use one of the criteria that adjust for the dimension—mBIC, EBIC, mAIC, or EAIC—possibly with a measure of the effective number of tests, and preferentially the EAIC if a specific level of FWER is targeted.

## Conflicts of Interest

The authors declare no conflicts of interest.

## Supporting information



Supporting information.

**Data S1.** Supporting information.

## Data Availability

We share our simulation code in the supporting information. The pharmacovigilance data provided by the ANSM are not freely available for privacy reasons.
