# Supplementary material for: A Simple Information Criterion for Variable Selection in High‐Dimensional Regression
Source: Stat Med. 2024 Dec 12;44(1-2):e10275. doi: 10.1002/sim.10275 (PMC11702156; doi:10.1002/sim.10275)
Supplement: Supplementary file 1 — Supporting information. [file SIM-44-0-s002.pdf]

A simple information criterion for variable selection in high-dimensional regression — Supporting information

1 Proof of the FWER-bounding property

We assume that each Δl^i follows its asymptotic distribution: a gamma distribution with probability density $f(x) = \frac{1}{\sqrt{\pi}} x^{-\frac{1}{2}} e^{-x}$. Let us prove that the associated cumulative distribution function, F, has the following bounds:

$$1 - f(x) \leq F(x) \leq 1 - \left(1 - \frac{1}{2x}\right) f(x) \quad (*)$$

Let $G(x) = 1 - f(x)$ and $H(x) = 1 - \left(1 - \frac{1}{2x}\right) f(x)$. Since f has limit 0, both G and H have limit 1 in $+\infty$. Therefore for all x,

$$F(x) = 1 - \int_x^\infty F'(t)\, dt$$

$$G(x) = 1 - \int_x^\infty G'(t)\, dt$$

$$H(x) = 1 - \int_x^\infty H'(t)\, dt$$

Also,

$$G'(t) = -f'(t) = \left(1 + \frac{1}{2t}\right) f(t)$$

$$F'(t) = f(t)$$

$$H'(t) = -\frac{1}{2t^2} f(t) - \left(1 - \frac{1}{2t}\right) f'(t)$$

$$= -\frac{1}{2t^2} f(t) + \left(1 - \frac{1}{4t^2}\right) f(t) = \left(1 - \frac{3}{4t^2}\right) f(t)$$

$$H' \leq F' \leq G'$$

By combining this inequality with the integrals above, $G(x) \leq F(x) \leq H(x)$. $(*)$ is proven.

Now we use $(*)$ to bound $\log F(x)$. We use the following inequality (a consequence of the log's Taylor series expansion at 1):

$$\forall t \in [0, \frac{1}{2}], -t - t^2 \leq \log(1-t) \leq -t$$

$$\forall x \geq 1, \log F(x) \geq \log\left(1 - f(x)\right) \geq -f(x) - f(x)^2$$

$$\log F(x) \leq \log\left(1 - \left(1 - \frac{1}{2x}\right) f(x)\right) \leq -\left(1 - \frac{1}{2x}\right) f(x)$$

$$-\left(1 + f(x)\right) f(x) \leq \log F(x) \leq -\left(1 - \frac{1}{2x}\right) f(x)$$

$$\log\left(1 - \frac{1}{2x}\right) \leq \log \frac{-\log F(x)}{f(x)} \leq \log\left(1 + f(x)\right)$$

We define:

$$T(x) = \log \frac{-\log F(x)}{f(x)}$$

By the inequalities above, $\lim_{x\to\infty} T(x) = 0$. Now we transform $T(x)$. By $f(x)$'s expression,

$$T(x) = \log\left(-\log F(x)\right) + x + \frac{1}{2}\log x + \frac{1}{2}\log \pi$$

For all q,

$$T(x) = \log\left(-\log F^q(x)\right) - \log q + x + \frac{1}{2}\log x + \frac{1}{2}\log \pi$$

For all q, q', α,

$$\begin{array}{rcl} T(x) & = & \log\left(-\log F^q(x)\right) \\ & + & \log q' - \log q \\ & + & \frac{1}{2}\left(\log x - \log\log q'\right) \\ & + & x - \log q' + \frac{1}{2}\log\log q' + \frac{1}{2}\log \pi \end{array}$$

By the definition:

$$x_{q',\alpha} = \log q' - \frac{1}{2}\log\log q' - \log\left(-\log(1-\alpha)\right) - \frac{1}{2}\log \pi$$

we have:

$$\begin{array}{rcl} T(x_{q',\alpha}) & = & \log\left(-\log F^q(x_{q',\alpha})\right) \\ & + & \log q' - \log q \\ & + & \frac{1}{2}\left(\log x_{q',\alpha} - \log\log q'\right) \\ & - & \log\left(-\log(1-\alpha)\right). \end{array}$$

And when q' goes to infinity,

$$\log\log q' - \log\left(-\log(1-\alpha)\right) - \frac{1}{2}\log\pi = o\left(\log q'\right)$$

$$x_{q',\alpha} \sim \log q'$$

$$\lim_{q'\to\infty} \log x_{q',\alpha} - \log\log q' = 0$$

Also,

$$\lim_{\substack{q,q'\to\infty \\ q\sim q'}} \log q' - \log q = 0$$

In the decomposition of $T(x_{q',\alpha})$ above, the second and third lines have limit zero when $q, q' \to \infty,\ q \sim q'$. $T(x_{q',\alpha})$ also has limit zero. Therefore:

$$\lim_{\substack{q,q'\to\infty \\ q\sim q'}} \log\left(-\log F^q(x_{q'})\right) = \log\left(-\log(1-\alpha)\right)$$

$$\lim_{\substack{q,q'\to\infty \\ q\sim q'}} F^q(x_{q'}) = 1-\alpha.$$

2 Supplementary simulation results

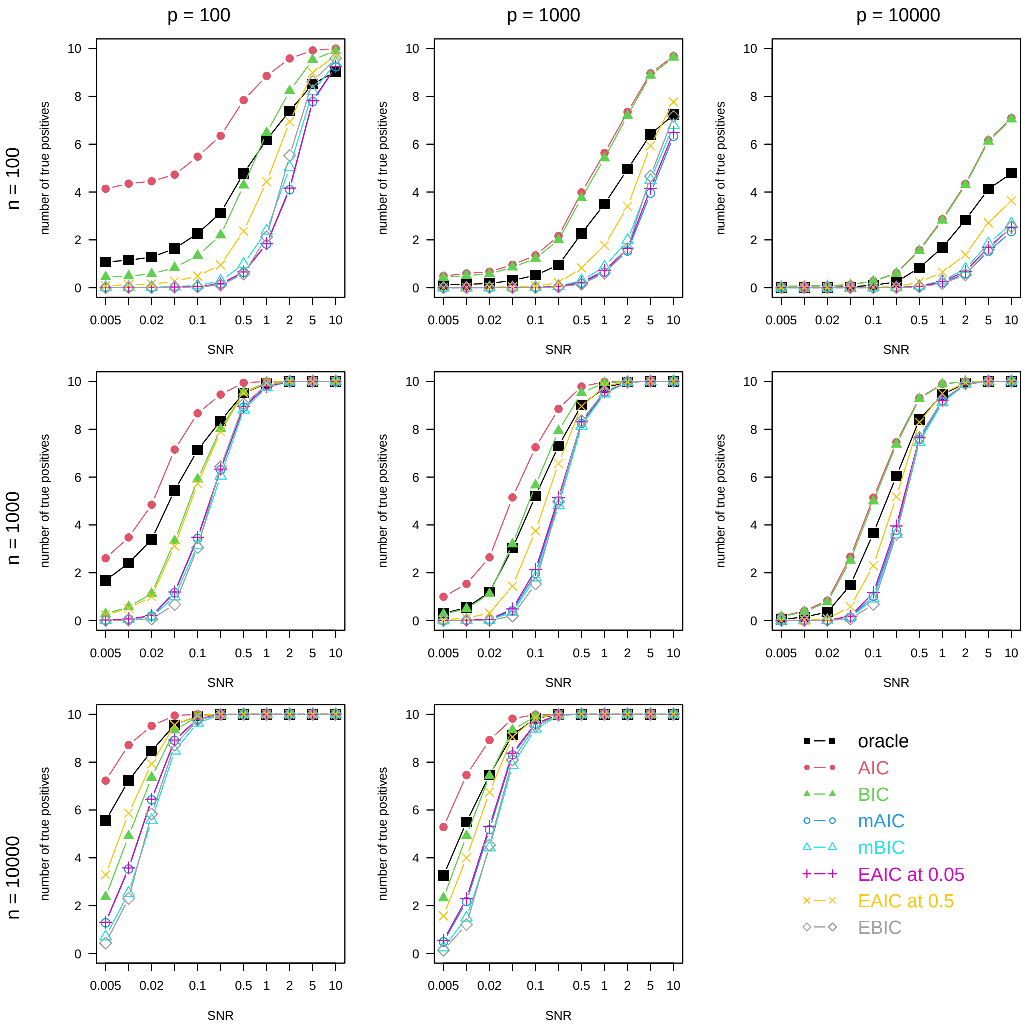

Figure S1: Full procedure simulation study: number of true positives by setting, averaged on 1000 simulations. Linear model, $\rho = 0$.

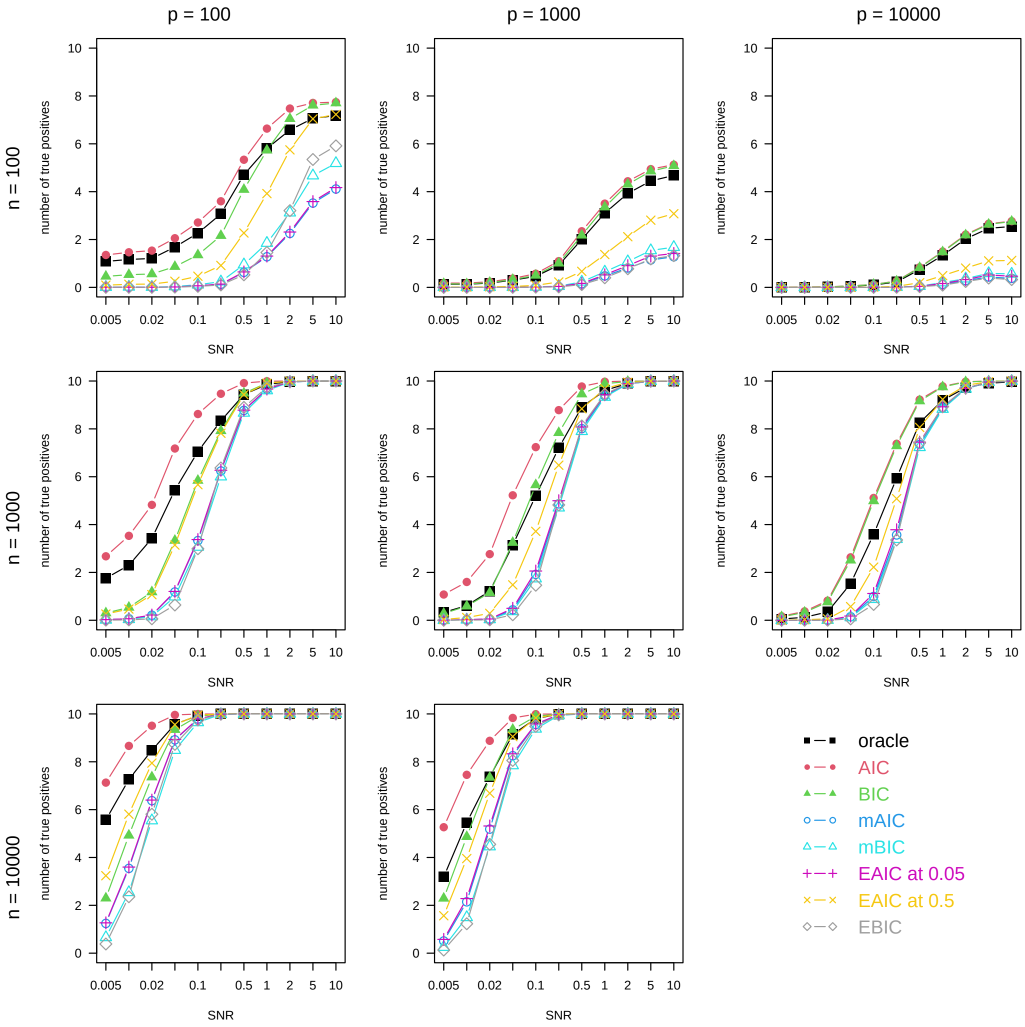

Figure S2: Full procedure simulation study: number of true positives by setting, averaged on 1000 simulations. Logistic model, $\rho = 0$.

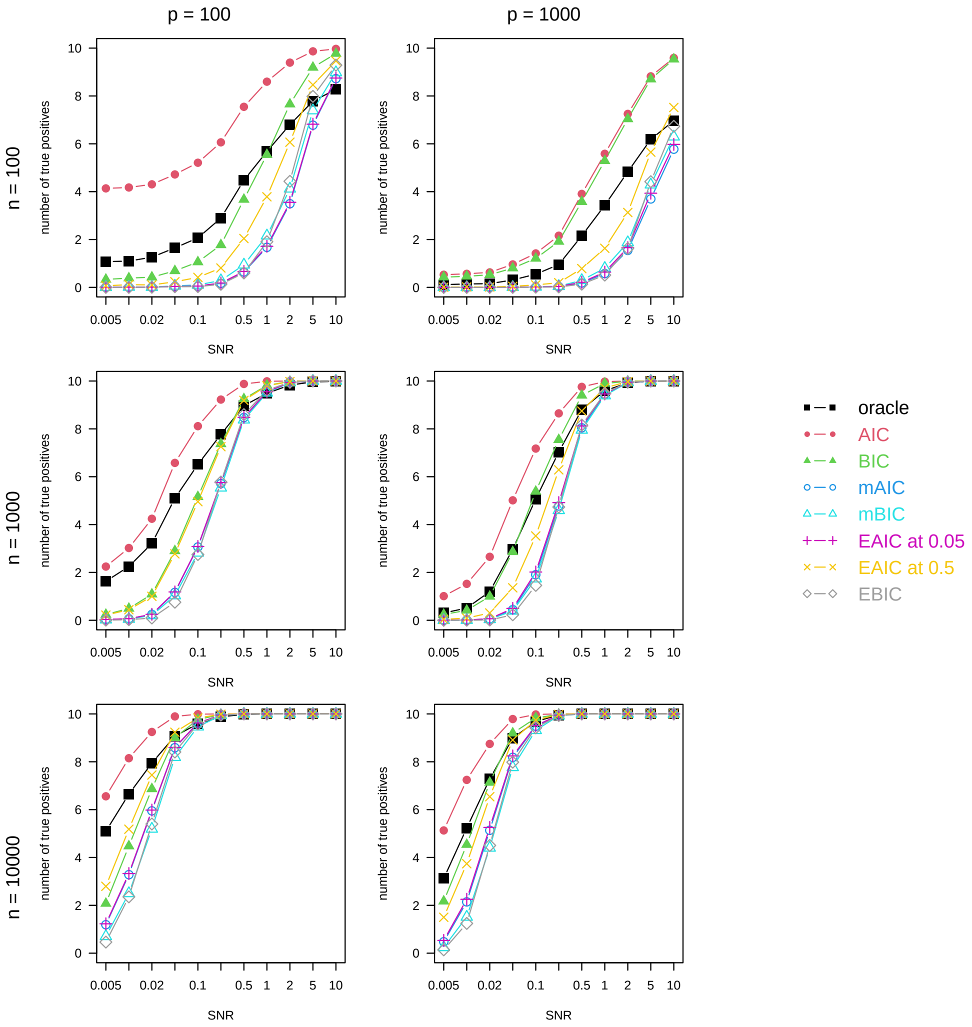

Figure S3: Full procedure simulation study: number of true positives by setting, averaged on 1000 simulations. Linear model, $\rho = 0.5$.

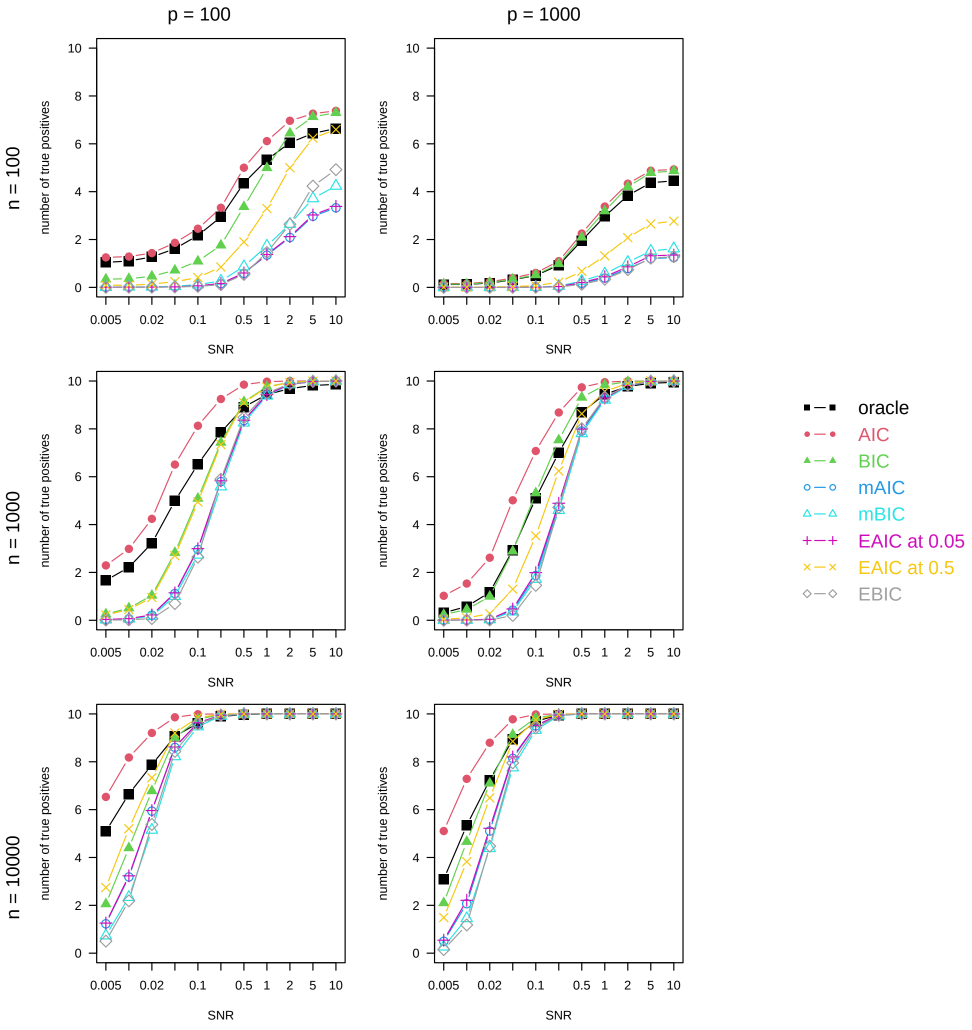

Figure S4: Full procedure simulation study: number of true positives by setting, averaged on 1000 simulations. Logistic model, $\rho = 0.5$.

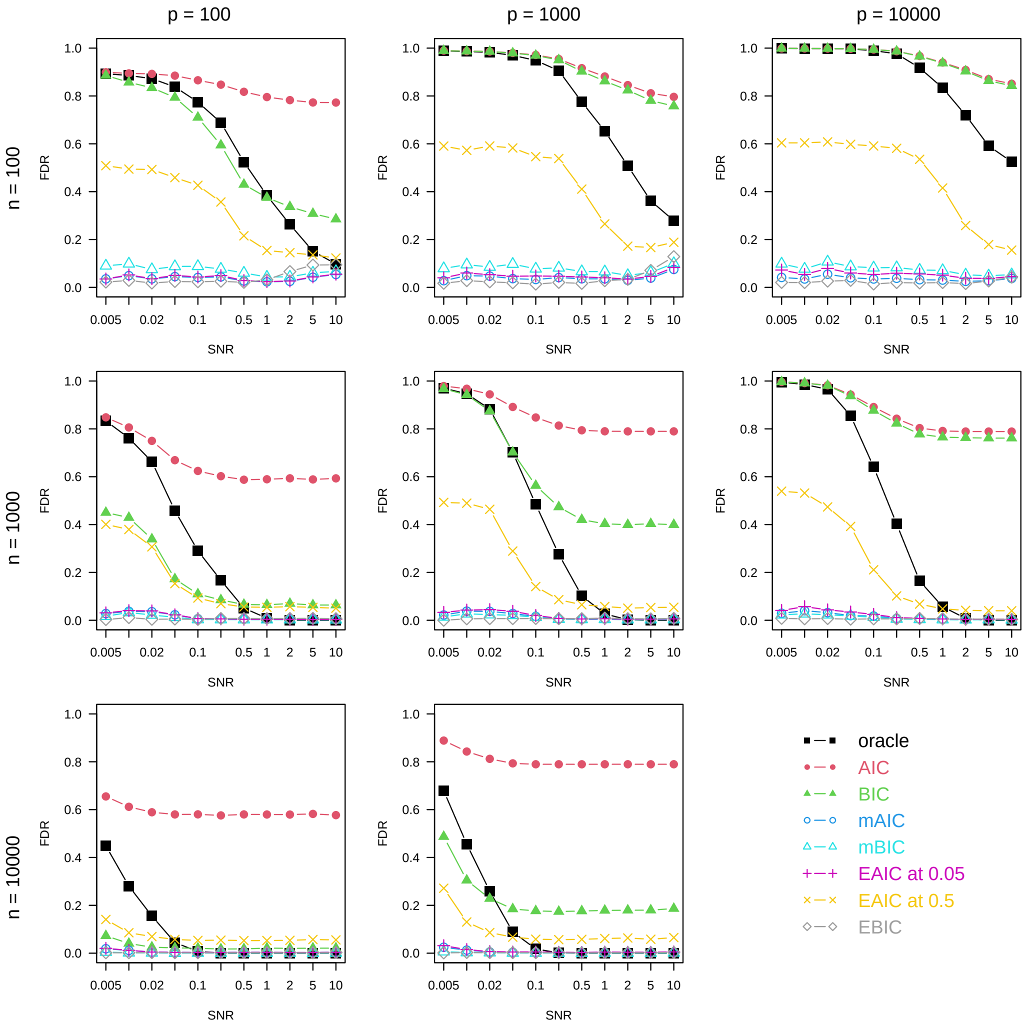

Figure S5: Full procedure simulation study: FDR by setting, averaged on 1000 simulations. Linear model, $\rho = 0$.

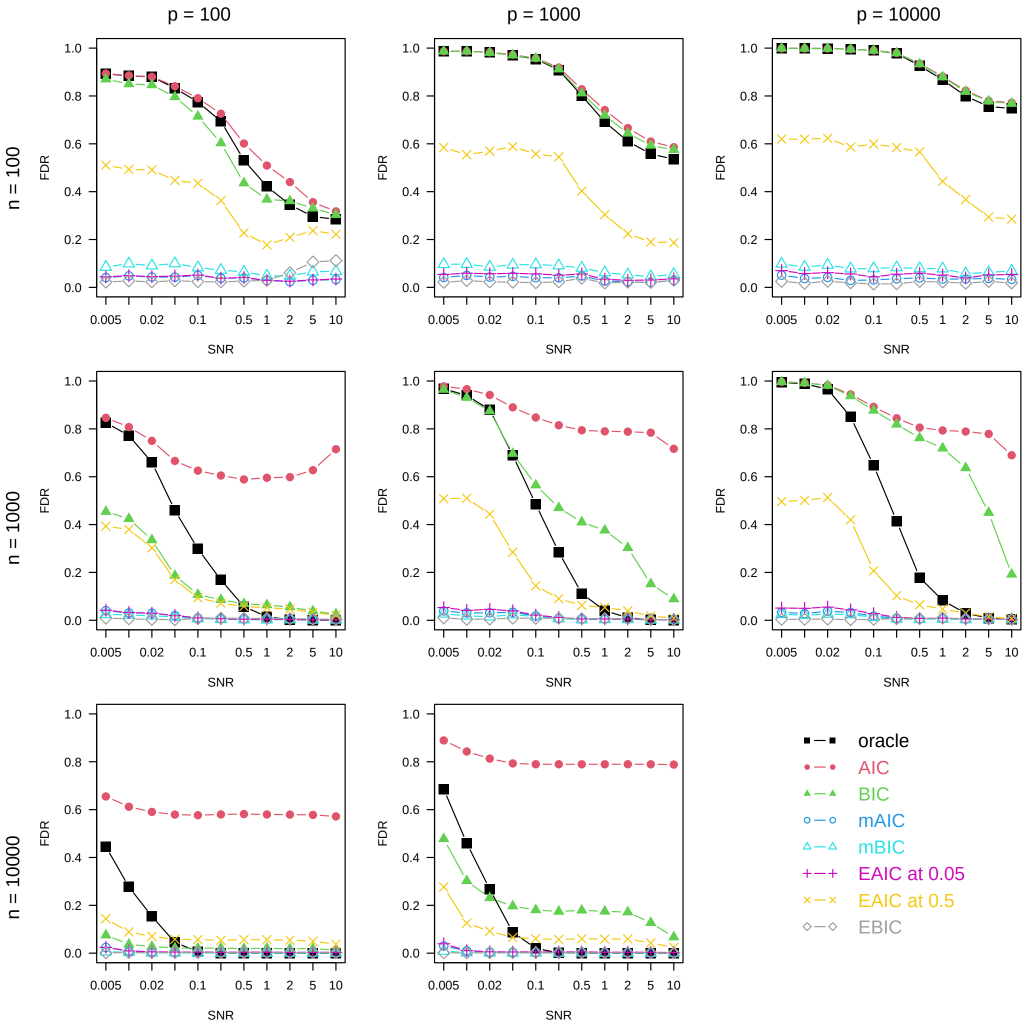

Figure S6: Full procedure simulation study: FDR by setting, averaged on 1000 simulations. Logistic model, $\rho = 0$.

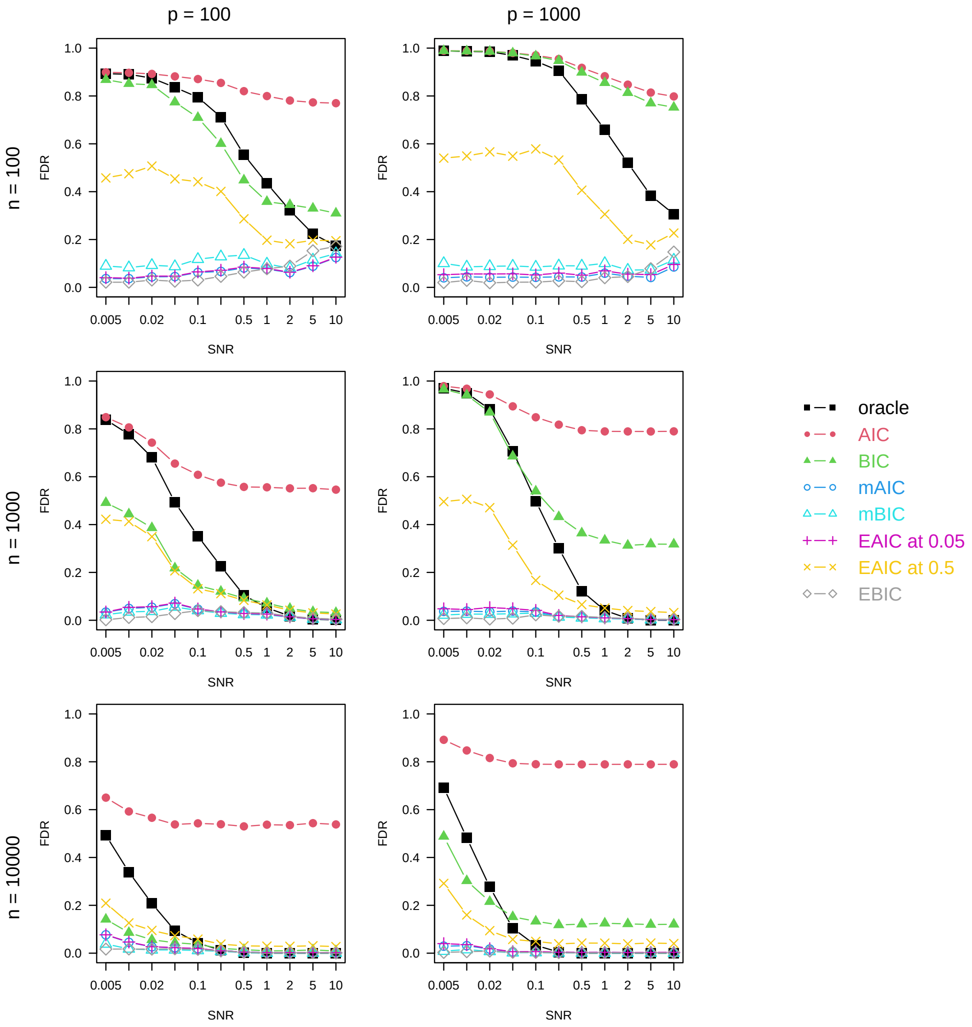

Figure S7: Full procedure simulation study: FDR by setting, averaged on 1000 simulations. Linear model, $\rho = 0.5$.

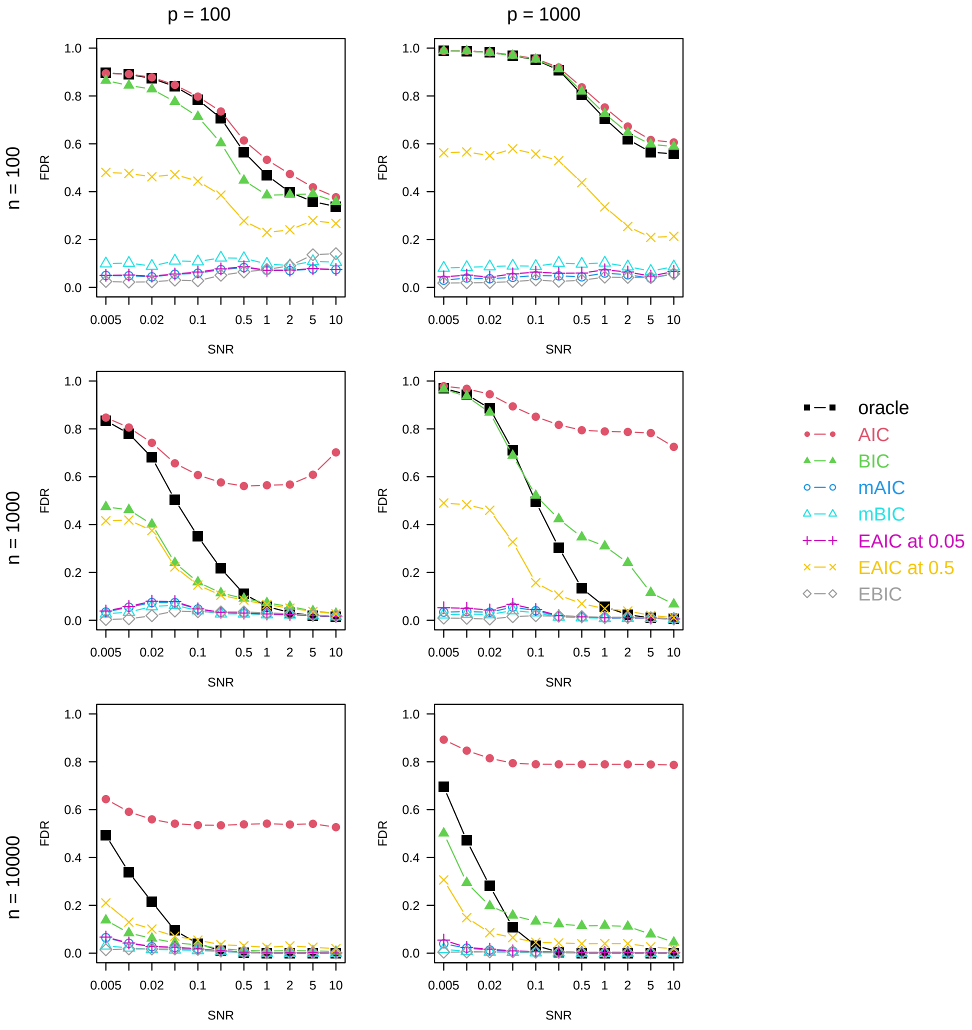

Figure S8: Full procedure simulation study: FDR by setting, averaged on 1000 simulations. Logistic model, $\rho = 0.5$.
